# Predictors and persistence of foot problems in older women: a six year prospective study

**DOI:** 10.1186/1757-1146-4-S1-O30

**Published:** 2011-05-20

**Authors:** Hylton B Menz, Elizabeth LM Barr, Wendy J Brown

**Affiliations:** 1 Musculoskeletal Research Centre, Faculty of Health Sciences, La Trobe University, Bundoora, Victoria 3086, Australia; 2 Baker IDI Heart and Diabetes Institute, Melbourne, Victoria 3004, Australia; 3 School of Human Movement Studies, Faculty of Health Sciences, University of Queensland, St Lucia, Queensland 4067, Australia

## Background

Foot problems are common in older people, particularly older women. However, no prospective studies have been undertaken to determine the incidence of foot problems, or the extent to which foot problems resolve or persist over time. Therefore, the aim of this study was to examine the prevalence and correlates of foot problems in older women over a six year period.

## Methods

Women aged 70 to 75 years who participated in the Australian Longitudinal Study on Women’s Health completed a postal questionnaire incorporating questions relating to demographics, major medical conditions and health status in 1999 (n=8,059) and 2005 (n=4,745). Key variables explored included self-reported foot problems, major medical conditions and body mass index (BMI).

## Results

At baseline, 26% of the sample reported foot problems. At follow-up, 37% remained free of foot problems, 36% had developed a new foot problem, 13% experienced resolution of their foot problems and 14% experienced persistent foot problems. Increase in BMI over the six year follow-up period was significantly associated with the development of new foot problems and the persistence of existing foot problems.

## Conclusions

Foot problems are common in older women and are associated with increased BMI. Nearly half of all older women without foot problems will report a new foot problem over a six year period, and in those with existing foot problems, approximately half will resolve and half will persist. Maintaining a healthy bodyweight may play a role in the prevention of foot disorders in older women.

